# Multifocal giant cell tumor of the carpus: Unusual presentation. Case report and review of the literature

**DOI:** 10.1016/j.ijscr.2023.109127

**Published:** 2023-12-08

**Authors:** Luis Carlos Gómez Mier, Camilo Soto Montoya, Andrea Franco Betancur, Juan Fernando Chaustre, Andres Felipe Ramírez, Sergio A. Arroyave Rivera

**Affiliations:** a Department of Orthopaedic Oncology, National Cancer Institute, Bogotá 111511, Colombia; b Department of Orthopaedic Oncology, Universidad Militar Nueva Granada, Bogotá 11011, Colombia; c Department of Orthopaedic Oncology, National Cancer Institute, Bogotá 11011, Colombia

**Keywords:** Giant cell tumor, Carpus, Hamate, Triquetrum, Arthrodesis, Case report

## Abstract

**Introduction and importance:**

Giant cell tumors (GCTs) of bone in the carpus are rare. Carpal GCTs are usually solitary lesions; multifocal involvement is exceptional. These lesions have a higher risk of local recurrence after intralesional curettage than those in other body areas.

**Case presentation:**

We present a case of a 28-year-old male with a six-month history of a palpable mass in the dorsal aspect of the left wrist. Physical examination revealed a 2 cm, mildly tender mass. Magnetic resonance revealed a large intermediate signal lesion involving completely hamate bone and the distal portion of the triquetrum. Histological examination confirmed a giant cell tumor of the carpus. The patient underwent en-bloc resection of the hamate bone extending to the distal part of the pyramidal. The defect was reconstructed using polymethylmethacrylate cement (PMMA), and intercarpal arthrodesis with the capitate was achieved. Follow-up at 18 months revealed a good clinical evolution, wrist range of motion of 30° of extension, 30° of flexion, and 10° of ulnar and radial deviation without evidence of tumoral recurrence.

**Clinical discussion:**

The current literature suggests a high incidence of local recurrence in carpal GCT, so wide excision with carpal arthrodesis is recommended, especially in Campanacci III and multifocal involvement.

**Conclusion:**

Carpal GCT is exceptional, mainly affecting the hamate, capitate, and scaphoid. Most literature supports wide excision of carpal GCT owing to the high recurrence rate with intralesional procedures.

## 1 Introduction

Giant cell tumor (GCTs) accounts for 5 % of primitive bone tumors, and are benign but locally aggressive [1]. GCT is commonly diagnosed in patients between 20 and 45, with a slight female predilection [2,3].

This rare entity usually arises in the metaphysis of long bones of skeletally mature patients, more than 50 % located around the knee [4].

The hand location is extraordinary. The primary involvement in this site is the metacarpals and phalanges [5]. Only 0.2 % of GCTs are localized in the carpal bones, and 60 % affect capitate and hamate bones [6]. This low incidence often makes physicians not suspect GCT in carpal bones [7].

These lesions have a higher risk of local recurrence (60 %–87 %) after intralesional curettage than those presenting in other body areas [8]. Therefore, some surgeons recommend wide resections and reconstruction with arthrodesis or row carpectomy in cases of cortical erosion or multifocal involvement [9].

There are currently few reports in the literature about simultaneous involvement in two or more bone giant cell tumors in the carpus.

The purpose of this report is to present a case of multifocal GCT of bone involving the hamate and triquetrum with soft tissue extension that was treated by en-bloc tumor resection and reconstructed with intercarpal arthrodesis and augmentation with polymethylmethacrylate cement.

Written informed consent was obtained from the patient for publication of this case report and accompanying images. The work has been reported in line with the SCARE criteria [10].

## 2 Case presentation

A 28-year-old, right-handed male presented with pain over the dorsal-ulnar level of the left wrist for six months. There was no history of previous trauma. He denied any personal or family history of cancer.

The examination revealed a predominantly dorsal-ulnar carpal mass adhered to deep planes without cutaneous changes. It measured approximately 2 cm in both horizontal and longitudinal dimensions. The patient complained about activity-related pain, especially during dorsiflexion (5/10 in the Visual Analog Score (VSA)). The grip strength was diminished by 50 % compared to the unaffected side.

The initial radiological work-up with plain radiography showed an expansile lytic lesion in the hamate, with thinned cortices and doubtful compromise of the triquetrum bone.

**Fig. 1:**
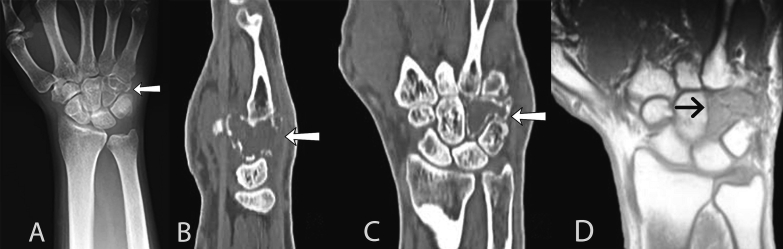
(a) Preoperative anteroposterior radiograph of the wrist showing expansile lytic lesion of the hamate. (b) Coronal and (c) sagittal sections of computed tomography show a lytic lesion at the hamate bone, expansile, loss of the intraosseous trabecular pattern, and cortical disruption in the dorsal bone surface. (d) The coronal section of T1-weighted MRI confirmed tumor extension to the distal one-third of the triquetum bone showing the other carpal bones unaltered.

Computed tomography of the wrist showed a loss of the trabecular pattern of the hamate, cortical thinning, and dorsal cortical disruption associated with soft tissue mass, classifying it as a Campanacci III tumor. The tumor measured 22 × 18 millimeters in width-length in coronal images. Magnetic resonance imaging (MRI) revealed a lesion involving completely hamate bone and distal portion of the triquetrum, displaying isointensity to the surrounding muscle on T1-weighted imaging and heterogeneous high intensity on T2-weighted fat-suppression imaging (Fig. 1).

The extension study was completed with chest tomography, ruling out metastatic lung involvement.

A percutaneous needle biopsy was performed dorsally. The histological analyses revealed a highly cellular lesion composed of nonneoplastic osteoclast-like giant cells, between which mononuclear neoplastic cells were embedded with some areas of hemorrhage A reactive rim of woven bone replaced cortical bone at the tumor periphery.

## 3 Surgical technique

The patient was placed on the operating table in the supine position. A long dorsal wrist incision followed a guideline between the fourth and fifth metacarpals. The extensor tendons were exposed, and a dorsal carpal arthrotomy was performed. Intercarpal ligaments between the hamate and capitate and carpal metacarpal ligament between the hamate and fourth and fifth metacarpals were sectioned.

Treatment consisted of en-bloc resection of the hamate and distal one-third of the triquetrum. The adjacent ulnar articular surface of the capitate, cartilage of the base of the fourth and fifth metacarpal bones, and middle third of the pyramidal bones were resected using a high-speed burr and argon beam coagulation as an adjuvant agent.

The proximal one-third of the triquetrum was preserved after careful inspection to confirm the lack of involvement of the remaining bone.

**Fig. 2:**
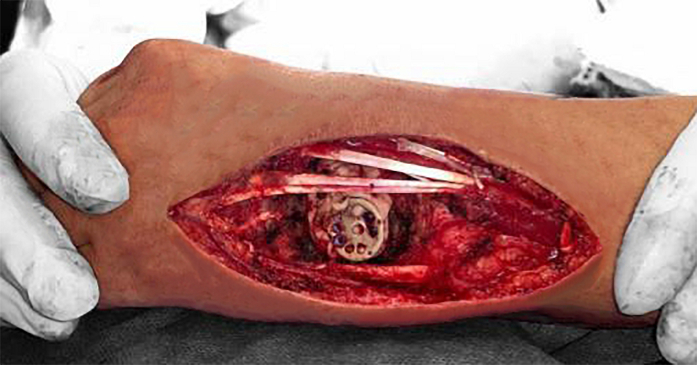
Image of the surgical intervention showing the dorsal wrist approach and the placement of four corner arthrodesis miniplate for intercarpal fixation.

**Fig. 3:**
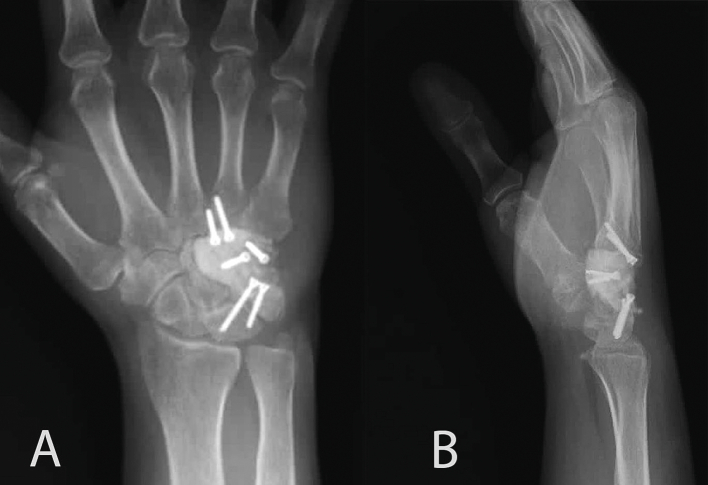
(a) Postoperative anteroposterior and (b) sagittal radiographs of the wrist showing final reconstruction with polymethylmethacrylate cement (PMMA) and a four-corner wrist arthrodesis mini plate.

The defect was reconstructed using polymethylmethacrylate cement (PMMA), and fixation was achieved with a four-corner wrist arthrodesis miniplate with screws directed to the lunate and fourth metacarpal base (Fig. 2, Fig. 3).

**Fig. 4:**
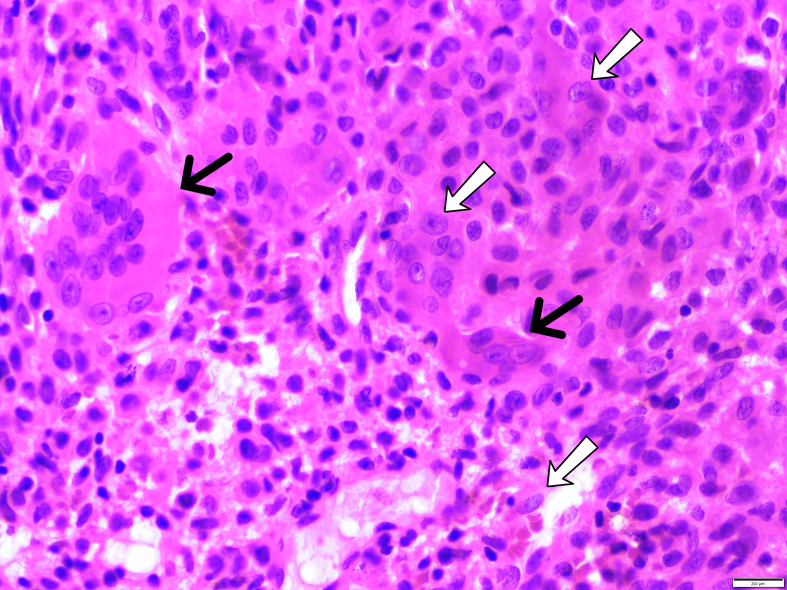
Histological photomicrograph (hematoxylin-eosin stain) of the en-bloc specimen. Osteoclast-like giant cells (black arrows) and mononuclear neoplastic cells exhibit a variety of morphological appearances, including rounded, oval, and spindled cells (white arrows).

The gross appearance of the tumoral lesion was soft and slightly brownish tissue. Specimen histologic examination confirmed the diagnosis of GCT (Fig. 4).

**Fig. 5:**
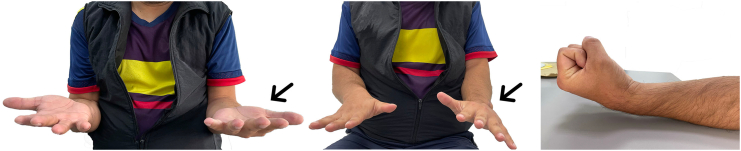
Demonstration of range of motion 18 months after surgery. Complete pronosupination and acceptable flexo-extension (black arrows marking the affected side) of the wrist with no evidence of local recurrence.

The postoperative period was uneventful, with the joint being immobilized with forearm-palmar plaster for six weeks. The patient reported an 80 % pain improvement according to the preoperative VSA score. Eighteen months after the intervention, the patient presented a good clinical evolution without pain or radiological signs of tumor recurrence. The grip strength of the left hand was 80 % of that of the unaffected side. Physical examination revealed a wrist range of motion of 30° of extension, 30° of flexion, and 10° of ulnar and radial deviation (Fig. 5). He was already reintegrated into his work activities with slight limitations in daily living activities.

## 4 Discussion

We present a case of a 28-year-old male with a carpus GCT. The uniqueness of this case is based on carpus location and multifocal involvement, which has been described in a few reports in the current literature.

Giant cell tumors represent benign but locally aggressive conditions. Its presence at the hand level is limited to 2 % to 4 % of all cases, mainly affecting the metacarpals and phalanges [11].

Despite its benign nature, it is known that metastasis of GCT can occur in distant organs in approximately 2 % of patients, with the lung being the most common site. Some literature suggests that acral and atypical locations of GCTs are prone to metastasize more frequently than tumors of the usual areas around the knee [3,12].

Carpal GCT is exceptional, most of them affecting hamate (31 %), capitate (24 %), and scaphoid (14 %), with the least frequent involvement being in the pyramidal tract, trapezius, and trapezoid [5,13].

GCT usually presents as a solitary lesion; multicentric and multifocal GCT is characterized by more than one primary GCT lesion and accounts for approximately 1 % of GCT cases [7]. Multifocal involvement in carpal GCTs is extremely rare [14].

Traditionally, intralesional curettage with local adjuvant methods has been the preferred treatment method for GCT, irrespective of tumor location [4]. The decision between intralesional and wide resections is based on considering an equilibrium between the risk of local recurrence and the preservation of segment function.

Intralesional curettage of the GCT in the metacarpals and carpal bones was associated with a high incidence of local recurrence [15,16] and there is no consensus regarding the ideal treatment of these lesions in hand locations.

Howard and Lassen [9] evidenced a high incidence of recurrence in carpal GCTs. They recommended resection of the carpus with intercarpal arthrodesis if the distal row was involved or proximal row carpectomy if the scaphoid or lunate was involved.

We chose en-bloc resection based on the local extension, hand location (high risk of recurrence), and extraosseous involvement (Campanacci grade III).

We performed distal and partial row carpectomy with intercarpal arthrodesis with capitate and fixation to the base of the fourth and fifth metacarpals. The choice of PMMA augmentation instead of autograft reconstruction was based on the high risk of local recurrence, which is radiologically easier to identify with PMMA than autograft or other void fillers.

Regarding the cases of multifocal carpal GCTs, there is little experience in the literature. We found four previous reports showing simultaneous involvement of multiple carpal bones (Table 1).

**Table 1:** Summary of published cases of multifocal carpal giant cell tumors. ROM: Range of motion.

Study	Year	Patients (n)	Gender and age (years)	Bones	Surgical treatment	Reconstruction after resection	Follow-up and final ROM	Recurrence
Gupta et al.	1995	1	Male 30	Capitate, hamate and triquetrum	Wide excision Distal row carpectomy	Bicortical iliac autogenous bone graft	18 months ROM not described.	No
Tarng et al.	2009	1	Female 29	Trapezium, trapezoid, capitate, and scaphoid	Intralesional curettage	Bicortical iliac autogenous bone graft	12 months 40° of extension and 50° of flexion.	No
Abdusamad et al.	2020	1	Female 29	Capitate and hamate	Wide excision	Triicortical iliac autogenous bone graft	18 months ROM not described.	No
Ansari et al.	2014	1	Male 14	Capitate, hamate, trapezium and base of third metacarpal	Wide excision	Bicortical iliac and fibular autogenous bone grafts	16 weeks 40° of extension and 30° of flexion.	No

Gupta et al. [17] reported a case of a multifocal GCT of the carpus affecting the hamate, capitate, trapezium, and trapezoid. The patient underwent wide excision with distal row carpectomy and reconstruction of the carpus with iliac bone graft. At the final follow-up, there were no recurrences. Tarng et al. [15] described a case involving the trapezium, trapezoid, capitate, and scaphoid with soft tissue extension. They performed intralesional curettage and reconstruction with autogenous corticocancellous iliac crest bone graft. Successful union with no recurrence was noted at the 1-year follow-up.

The most recent report was published by Abdusamad et al. [18]. They reported a case of a 29-year-old female with simultaneous involvement of capitate and hamate. They performed wide resection, filling the defect with an autologous bone graft. Follow-up at 18 months demonstrated no evidence of local recurrence.

Ansari et al. [14] reported a multifocal GCT of carpal bones in a 14-year-old male. Interestingly, this is the only description of multifocal GCT in a skeletally immature patient. Similar to our case, they performed en-bloc resection via a dorsal approach and intercarpal arthrodesis with iliac crest autograft. Solid union was noted 16 weeks after surgery, and good function was achieved.

Ours is the fifth case with multifocal involvement of carpal bones. At the final follow-up, the patient reached good function. The grip strength of the left hand was 80 % of that of the unaffected side, and the physical examination revealed an almost complete wrist range of motion. At eighteen months postoperatively, there were no radiological signs of tumor recurrence.

## 5 Conclusion

The limited number of cases of GCT with multifocal carpal bone involvement and the heterogeneity in the published treatments represent a therapeutic challenge in these neoplasms. Currently, most literature supports the choice of wide excision of giant cell tumors of bone involving carpal bones owing to the high recurrence rate with intralesional procedures. Partial row carpal arthrodesis is a reasonable option in multifocal tumor involvement with good functional and oncological outcomes.

## Consent

Written informed consent was obtained from the patient for publication of this case report and accompanying images. A copy of the written consent is available for review by the Editor-in-Chief of this journal on request.

## Ethical approval

This study was approved by the institutional review board.

## Funding

This research did not receive any specific grant from funding agencies in the public, commercial, or not-for-profit sectors.

## CRediT authorship contribution statement

All authors participated significantly in the development of this work. CSM and LCGM conceived the study idea. SAAR, JFC, AFR, and AR coordinated the data collection. SAAR drafted and edited the manuscript. All authors read and approved the final manuscript.

## Guarantor

Sergio A. Arroyave Rivera.

## Declaration of competing interest

The authors have no conflict of interest to declare.

## Data Availability

The data that support the findings of this study are available from the corresponding author upon reasonable request.
